# The effects of long-term fertilizations on soil hydraulic properties vary with scales

**DOI:** 10.1016/j.jhydrol.2020.125890

**Published:** 2021-02

**Authors:** Xiaoxian Zhang, Andrew L. Neal, John W. Crawford, Aurelie Bacq-Labreuil, Elsy Akkari, William Rickard

**Affiliations:** a Department of Sustainable Agricultural Sciences, Rothamsted Research, Harpenden AL5 2JQ, UK; b Sustainable Agriculture Sciences, Rothamsted Research, North Wyke EX20 2SB, UK; c Adam Smith Business School, University of Glasgow, West Quadrangle, Glasgow G12 8QQ. UK; d Division of Agriculture & Environmental Sciences, School of Biosciences, University of Nottingham, Sutton Bonington Campus, Leicestershire LE12 5RD, UK

**Keywords:** Rothamsted long-term experiment, Hierarchical soil structural change, Pore-scale simulations, X-ray computed tomography

## Abstract

Soil structure is an indicator of soil quality and its alterations following cropping system conversion or fertilization change evolve slowly. How such alterations vary with scale remains elusive. We investigated this based on the Rothamsted long-term wheat experiment (since 1843) in the UK. Triplicate cores 7 cm high and 10 cm in diameter were taken from plots that have been under different fertilizations or returned to natural woodland for more than one century for imaging using X-ray computed tomography with the voxel size being 40 µm. We then broke each core and sampled three aggregates from it to scan with the voxel size being 1.5 µm. For each core and aggregate sample, we calculated its pore size distribution, permeability and tortuosity. The results showed that the fertilization change >170 years ago reshaped the soil structure differently between the core scale and the aggregate scale. Macro-porosity of the pores (>40 µm) in the cores unfertilized or fertilized with inorganic fertilizers was low and the pores were poorly connected in the top 10 cm of soil, compared to those given farmyard manure or in the woodland. In all treatments, the pores in the core images were hydraulically anisotropic with their permeability in the horizontal direction being higher than that in the vertical direction, whereas the aggregates were comparatively isotropic. The fertilization affected image porosity and permeability at core scale more significantly than at aggregate scale, and the aggregates fertilized with farmyard manure and in the woodland were more permeable than the aggregates in other treatments. It was also found that compared to no-fertilization or fertilization with complete fertilizers, fertilizing without phosphorus over the past 20 years increased the porosity and permeability of the aggregates but not of the cores. Fertilization with inorganic fertilizers increased the tortuosity of the macropores in the cores but not of the intra-aggregate pores, compared to no-fertilization. Porosity-permeability relationship for aggregates unfertilized or fertilized with inorganic fertilisers followed a power law with *R*^2^ > 0.8. In contrast, the permeability of the aggregates in farmyard manure and in the woodland trended differently as their porosity increased. The results also revealed that the transport ability of the aggregates and cores responded differently to carbon in that with soil carbon increasing, the permeability of the aggregates increased asymptotically while the permeability of the cores, especially its horizontal component, increased exponentially.

## Highlights

• Soil samples were taken from field under different fertilizations for 175 years.
• Soil permeability was calculated using X-ray tomography and pore-scale simulation.
• Soil was more isotropic and homogenous at aggregate scale than at core scale.
• Soil permeability at core scale increased exponentially with soil carbon.
• Soil permeability at aggregate scale increased asymptotically with soil carbon.

## 1 Introduction

Most soils in natural and managed terrestrial ecosystems are hierarchically structured with their pores ranging from less than one micron to several millimetres in diameter (Young and Crawford, 2004). Such structures are a result of the interplay of a multitude of abiotic and biotic processes operating over a wide range of scales, and are the foundation of all life in terrestrial ecosystems as they keep the soil moist and oxygenated (Young et al., 2008b). Macropores in soil are formed mainly by actions of plant roots, earthworms, aggregations, as well as swelling and shrinkage (Bronick and Lal, 2005), providing preferential pathways for water and nutrients to flow (Beven and Germann, 2013). In contrast, micropores formed biotically and abiotically function as storage to retain water and nutrients (Feeney et al., 2006, Kallenbach et al., 2016, Totsche et al., 2018). Microorganisms play a critical role in soil genesis and their feedback reactions with soil structure are mediated by organic matter in complex ways (Young et al., 2008a). However, it is generally accepted that boosting microbial activities increases soil porosity due to the enhanced soil aggregation. For example, Crawford et al (2012) found that incubating a repacked fine-textured soil for three weeks resulted in a 17% increase in porosity. The importance of soil structure in physical and biogeochemical processes is fairly understood. In addition to its well-documented role in controlling water flow and solute transport, soil structure also plays a critical role in other soil functions (Kravchenko and Guber, 2017) as recent findings revealed that enzymatic activities were strongly associated with a specific range of pores, implying that carbon and nutrient cycling in soil is also pore-dependant (Kravchenko et al., 2019)

Soil reorganizes its structure following agronomic practice changes (Caplan et al., 2017). Tillage and root growth could instantly reshape soil structure, while the change induced by microbial activities could be a slow process (Crawford et al., 2012, Kallenbach et al., 2016). For example, Bacq-Labreuil et al (2020) recently found that following conversion from a fallowed land to arable and grassland, the change in intra-aggregate structure did not reach equilibrium 10 years after the conversion; the Rothamsted long-term wheat experiment also revealed that soil carbon took approximately one century to stabilize after changing fertilization to farmyard manure (Poulton et al., 2018). As the dynamics of soil carbon is determined by its accessibility to microbes (Dungait et al., 2012) which in turn is modulated by soil structure, it is rational to conjecture that the change in soil structure following the fertilization change could not reach equilibrium before soil carbon stabilized. This is also corroborated by recent findings that soil physical properties affected soil carbon and nitrogen more than soil enzymes and microbial communities (Li et al., 2020). Understanding the multiscale soil structural alteration instigated by agricultural practice change is hence important as pores at different scales play different roles in hydrological and biogeochemical functions (Wang et al., 2019, Yudina and Kuzyakov, 2019).

Most macroscopic phenomena measured in soil emerge from biogeochemical and physical processes occurring at the microscopic scale (Baveye et al., 2018, Zhang et al., 2016), and it has been increasingly recognized that it is impossible to reliably predict physical and biochemical processes by studying the bulk soils alone (Wilpiszeski et al., 2019). As such, there has been a surge in study of soil structure and its impact on water and solute transport over the past two decades largely due to the development in X-ray computed tomography (CT) and its application in soil science (Helliwell et al., 2013, Huang et al., 2015, Schlüter et al., 2014). One application of X-ray CT is to quantify soil structural change induced by agronomic management (Lucas et al., 2019, Piccoli et al., 2019, Soto-Gomez et al., 2020), as well as its consequence for hydrological process (Armenise et al., 2018, Baveye et al., 2018, Gharedaghloo et al., 2018, Luo et al., 2010, Zhang et al., 2019, Zhou et al., 2019).

Modelling water flow and solute transport in hierarchically structured soils often separates their macropores and micropores into two hydraulically linked domains, with flow and transport in each domain represented by a set of equations and the mass exchange between the two domains described by empirical formulae (Gerke and van Genuchten, 1993, Larsson and Jarvis, 1999, Simunek et al., 2003, Vogel et al., 2000). Separating the pores into two domains improves the model but it also increases the numbers of parameters which have to be determined empirically by calibration against observed data (Frey et al., 2016, Haas et al., 2020, Lamy et al., 2009). The X-ray CT has potential to plug this gap but needs to scan the soil at multiscale so as to characterize the inter-aggregate pore geometry at a macro-scale and the intra-aggregates pore geometry at a micro-scale (Li et al., 2018b, Zhang et al., 2016). Linking soil hydraulic conductivity to pore geometry obtained from X-ray images is available, but most them integrated the macropores and micropores (Koestel et al., 2018, Pohlitz et al., 2019, Schlüter et al., 2020, Zhang et al., 2019). There are also studies on geometrical change in both intra-aggregate pores and macropores (Borges et al., 2019, Galdos et al., 2019, Koestel and Schlüter, 2019, Pires et al., 2019, Schlüter et al., 2018), as well as their temporal evolution under different managements (Lucas et al., 2019, Schlüter et al., 2011). While a change in agronomical practices could lead to an instant change in pore geometries, it might take decades or even century for the pore geometries to reach a new statistically equilibrium state on average across the field following fertilization change or land conversion (Lohse and Dietrich, 2005, Lucas et al., 2019, Neal et al., 2020a). Therefore, for experiments using repacked soils that did not last long enough (Crawford et al., 2012, Kallenbach et al., 2016, Menon et al., 2020, Rabbi et al., 2018), the observed soil structural change might be a just temporary transition rather than what the soil would ultimately evolve to.

The hierarchical soil structure is formed by a multitude of biotic and abiotic processes operating at different scales and how this multiscale structure responds to management practice change is poorly understood. Apart from this, another issue that has been overlooked is hydraulic anisotropy. Soil anisotropy is important not only in hillslope hydrology for lateral water flow but also in plant uptake of water as vertically-dominant roots drive water to flow mainly in the horizontal direction towards the roots (Zhang et al., 2020).

This paper aims to investigate the impact of long-term fertilizations on soil structural change and the associated hydraulic properties at different scales based on the Rothamsted long-term experiment in the UK that has been in operation since 1843. Soil samples taken from plots under different fertilizations for more than a century were scanned using X-ray CT at multiple scales, and the impact of the long-term fertilizations on soil structure was analysed based on the physical parameters calculated from these multiscale images.

## 2 Materials and methods

### 2.1 Site description

The Rothamsted long-term experiment at Broadbalk (Latitude 51° 48′ 34.44″ N; Longitude 0° 21′ 22.76″ W) started in 1843 to test the effects of various combinations of inorganic fertilizers (N, P, K and Mg) and organic manure on the yield of winter wheat, with a unfertilized strip as the control. The mean annual temperature and rainfall on the site is 10.1 °C and 701 mm respectively (http://www.era.rothamsted.ac.uk). The soil on the site is predominantly clay loam classified as Chromic Luvisol (FAO classification). The plough layer (0–23 cm) contains 25% of sand, 25% of silt and 50% of clay with an average particle density of 2.56 g/cm^3^ (Gregory et al., 2010). The pH is maintained at 7–7.5 by liming. Since its inception the experiment was made a few changes aimed to make it representative to the changes in farming in the UK. Detailed description of the experiment is available online (http://www.era.rothamsted.ac.uk) and in the literature (Blair et al., 2006, Watts et al., 2006). We provide in the Supplementary information the fertilization history and layout of the experimental site. In short, the site consists of 19 strips each associated with a specific fertilization ranging from farmyard manure to different combinations of nitrogen, phosphorus, potassium and magnesium minerals. Initially, all 19 strips were for continuous wheat and they all were made into 10 sections later aimed to compare the impact of other agronomic managements under the same fertilization, including a straw incorporation since1986. In 1882, a part on the west edge of the site was withdrawn from cultivation leading to development of a small area of woodland colonised by various grass species and trees, mostly Ash (Fraxinus excelsior), Sycamore (Acer pseudoplatanus) and Hawthorn (Craetagus monogyna) (Poulton et al., 2003).

### 2.2 Acquisition of soil images

Triplicate cores 7 cm high and 10 cm in diameter were taken in October 2015 from each of the following four plots (marked in the Supplementary information) that have been under different fertilizations since 1843: An unfertilized plot (referred to as CK), a plot given farmyard manure (referred to as FYM), and two other plots that have been fertilized with different combinations of inorganic minerals. There were some changes in fertilization after its inception and the details are given in the Supplementary information. In what follows we will refer the plot that is currently receiving 144 kg of N, 90 kg of K and 35 kg of P and 12 kg of Mg as N3, and the plot that has not received P since 2001 as No P. As a comparison, triplicate cores were also taken from the woodland. Each sample was taken by gently hammering a PVC core into the topsoil with the core-top 1–2 cm below the soil surface; we then dug the core out after removing the soil surrounding the core using a trowel. Loose soil and extra soil at the two ends of the core were peeled off using a sharp knife before tightly wrapping it with plastic films prior to x-ray imaging.

All cores were scanned using X-ray CT and details of the procedure were given in previous work (Bacq-Labreuil et al., 2018). In short, we used the Phoenix v∣tome∣x M scanner (GE Measurement and Control solution, Wunstorf, Germany) at the Hounsfield Facility of University of Nottingham to scan the cores under 160 kV and 180μA at a pixel resolution of 40 μm. After the scanning, each core was manually broken to pass through a series of sieves of 4, 2 and 0.71 mm by horizontally shaking at 300 rotations/min for 3 min, and three aggregates retained in the sieves of 2 mm and 0.71 mm were randomly selected and scanned using a Phoenix Nanotom® (GE Measurement and Control solution, Wunstorf, Germany) under 90 kV and 65μA at a pixel resolution of 1.51 μm. The sizes of all aggregates were thus approximately 0.8 mm to 4 mm. The scanned images were reconstructed using the software provided by the manufacturer with an optimisation to correct any possible sample-movement during the scanning process. To avoid internal collapse of the cores, all cores were scanned at prevailing water content approximately the field capacity, while the aggregates were scanned after an overnight of air-drying.

We first analysed the images using Image J with a region of interest (ROI) cropped out from each image for ease of analysis. The ROIs of the core images (1000x1000x1000) were positioned centrally to exclude the gaps between the soil and the PVC wall, while for the irregular aggregates we cropped a cuboid ROI (640x480x400) out of each image. The cropped ROIs were greyscale and they were segmented using the bin bi-level threshold method presented in Vogel and Kretzschmar (1996). Details of the segmentation method were given previously (Bacq-Labreuil et al., 2018). In short, 20 slices were randomly selected from each stack and each slice had a single threshold value that was calculated from the Li-threshold algorithm in Image J. The values of the two thresholds used in the bin bi-level method for segmenting the stack were calculated from the extreme values obtained from the 20 slices. Prior to pore geometry analysis and pore-scale simulation, all hydraulically isolated void voxels were removed and replaced by solid voxels.

### 2.3 Permeability

The permeability of all cores and aggregates was calculated from pore-scale simulation of water flow in their pore space using the lattice Boltzmann (LB) model we developed previously (Zhang et al., 2010). Most early LB models used for pore-scale simulation were based on the single-relaxation time (SRT) approach (Qian et al., 1992), but it was later found that the commonly used bounce-back method associated with the SRT model was erroneous for solving the fluid-wall boundary (Pan et al., 2006). The multiple relaxation time (MRT) LB method can resolve this problem (d'Humières et al., 2002) and was used in this paper. Details of the method and its implementation are given in the Supplementary information.

Water flow in each sample was driven by a pressure gradient in a direction generated by imposing a high pressure on one side and a low pressure on the opposite side of the sample; other four sides were treated as periodic boundaries. We selected three pressure gradients to ensure that water flow was laminar and that the average flow rate was proportional to the pressure gradient. In all simulations, the initial velocity was zero and the flow was simulated to steady state - deemed to have reached when the relative difference in the average flow rate at two moments spanning 100 time-steps was less than 10^-7^. We then sampled the velocity and pressure at all voxels and volumetrically averaged them over each cross-section perpendicular to the pressure gradient direction to calculate the permeability. We sampled the pressure aimed to check pore homogeneity as our previous work showed that the average pressure distribution could become nonlinear in highly heterogeneous soils (Li et al., 2018a). To test hydraulic anisotropy, we calculated the permeability in the three directions of each sample.

For simulation under each pressure gradient, the volumetric average water flow was assumed to follow the Darcy’s law. Taking the pressure gradient generated in the z direction as an example, that means

(1) $$q_{z}\left(z\right)=-\frac{k}{\rho u}\frac{\partial P}{\partial z},$$

where *q_z_*(*z*) is the average flow rate over the cross-section perpendicular to the *z* direction (cm/s), *k* is permeability (cm^2^), *u* is kinematic viscosity of water (cm/s), *P* is the averaged pressure over the cross-section (kPa), and ρ is water density (g/cm^3^). The average water flow rate and pressure were calculated from pore-scale simulation as follows:

(2) $$\begin{array}{c} q_{z}\left(z\right)=\sum_{i=1}^{N_{\mathrm{zw}}}u_{z}\left(x_{i},y_{i},z\right)/N_{z},\\ P\left(z\right)=\sum_{i=1}^{N_{\mathrm{zw}}}p\left(x_{i},y_{i},z\right)/N_{\mathrm{zw}}, \end{array}$$

where *N_z_* and *N_zw_* are the number of all voxels and the number of water-filled voxels in the cross section located at *z* respectively,$p\left(x_{i},y_{i},z\right)$and $u_{z}\left(x_{i},y_{i},z\right)$are water pressure and water velocity component in the *z* direction at voxel centred on $\left(x_{i},y_{i},z\right)$respectively.

At steady state$q_{z}\left(z\right)$is independent of z because of the mass balance constraint, and the permeability of the sample was hence calculated from

(3) $$k=\rho u\frac{q_{z}L}{P_{1}-P_{0}},$$

where *L* (cm) is the length of the sample in the *z* direction. Once the permeability *k* was calculated, its associated hydraulic conductivity *K* can be calculated from K = kg/μ, with *g* being the gravitational acceleration (cm/s^2^). Since water density and viscosity depend on temperature and chemical composition of the water, the hydraulic conductivity is not constant but varies with soil environment. In what follows, we hence use permeability rather than convert it to hydraulic conductivity.

### 2.4 Pore size distribution and tortuosity

We calculated the pore size distribution for both aggregates and cores using the Plug-in CT-image Analysis & Manipulation (SCAMP) (Houston et al., 2017) and Bone J in Image J respectively, finding that the difference between the two was less than 5%. In what follows we only present the results obtained from Bone J. We expressed the pore size distribution as volumetric fraction of pores of different sizes.

Tortuosity is a parameter widely used to characterize porous materials, but it could be calculated based on a specific transport process (Ghanbarian et al., 2013) or geometrical connection of the pores (Roque and Costa, 2020). Different methods could give different results. Since the focus of this paper is viscous fluid flow, following Koponen et al (1996), we calculated it as the ratio of the sum of the absolute bulk fluid velocity in all voxels to the sum of the velocity component in the pressure gradient direction. As soil permeability depends predominantly on hydraulically connected larger pores, for each sample we also calculated its critical pore diameter similar to that used in our previous work (Neal et al., 2020b). In brief, each pore mapped out by the Bone J comprised a group of void voxels. Starting from the pores with the largest diameter, we calculated if the void voxels in these pores could form a cluster(s) stretching from the inlet to the outlet faces. If they could not, we reduced the pore diameter sequentially until we found a diameter that the void voxels in pores with diameter equal to or larger than it formed a cluster (s) linking the inlet and the outlet faces.

## 3 Results

**Fig. 1:**
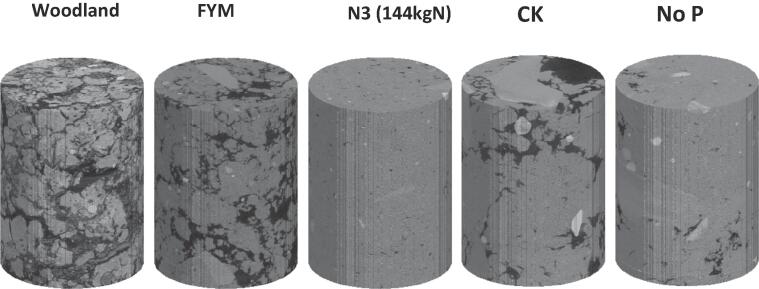
X-ray CT images of cores (40 μm resolution) taken from plots under different fertilizations and the woodland.

**Fig. 2:**
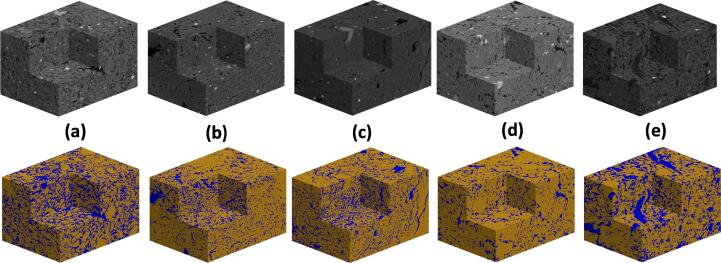
Representative greyscale (top panel) and segmented (bottom panel) images of the aggregates in cores taken from plots under different fertilizations and the woodland. (a) Farmyard manure, (b) N3, (c) No P, (d) CK, and (e) woodland.

Fig. 1 shows the greyscale images of the cores taken from the five treatments to visually illustrate their difference. Fig. 2 compares the greyscale images of the aggregates and their associated segmentation to show the impact of the treatments as well as the accuracy of the segmentation method. The sizes of the aggregates taken from the sieves varied, but no noticeable correlation was found between aggregate size and the parameters we calculated. In what follows, we hence pool the results calculated from the aggregates taken from the same treatment.

### 3.1 Pore geometries

**Fig. 3:**
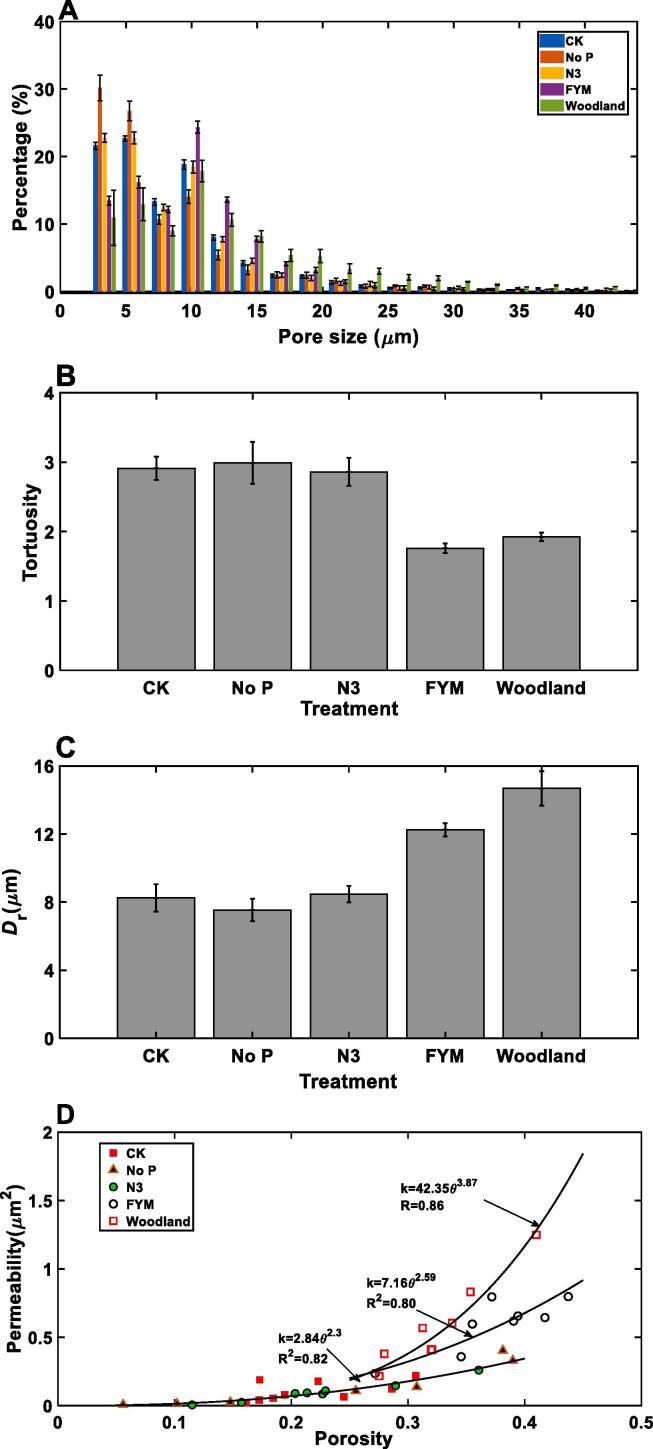
Pore-size distribution (A); tortuosity (B); (C) critical pore diameter; and (D) porosity–permeability relationship for aggregates in all treatments where the relationship for FYM and the woodland was fitted separately while those for non-fertilization and fertilization with inorganic fertilizers were pooled. The error bars represent standard errors.

Table 1 and Fig. 3A compare the porosity and pore-size distribution of the aggregates taken from soils under different treatments respectively. Overall, fertilizing with farmyard manure and the woodland increased the aggregate porosity, especially the proportion of pores >10 μm which was increased at the expense of pores smaller than10μm. The pores >17.5 μm were significantly higher in the woodland than in other treatments. Fig. 3B reveals that the porosity was negatively correlated to the tortuosity, with the tortuosity of the aggregates unfertilized or fertilized with inorganic fertilizers being close to each other but higher than that of the aggregates taken from the woodland and farmyard manure plots. The aggregates in the woodland and the farmyard manure plots also had much bigger critical pore diameters (12 μm and 14 μm respectively) than that taken from other plots (approximately 8 μm).

**Table 1:** Average and coefficient of variation (CV) of porosity and permeability of the aggregates under different treatments.

	Porosity		Permeability (μm^2^)	
	Mean	CV	Mean	CV
Woodland	0.331	0.129	0.568	0.545
FYM	0.377	0.119	0.595	0.295
N3	0.236	0.274	0.102	0.719
No p	0.248	0.405	0.146	1.149
Control	0.226	0.213	0.108	0.611

**Fig. 4:**
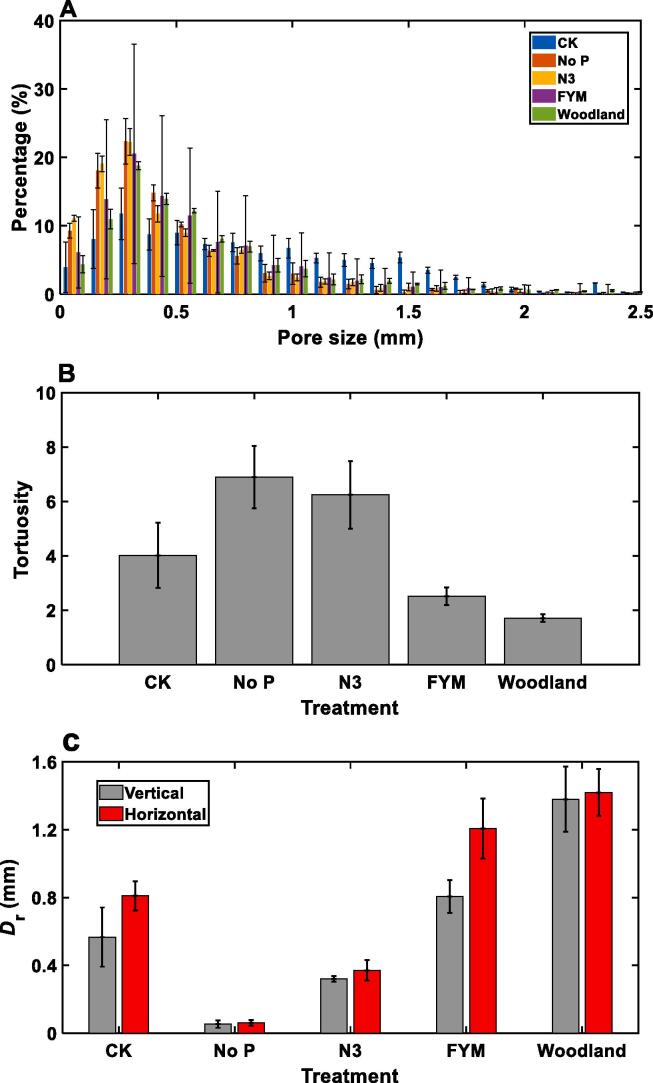
Pore-size distribution $\left(A\right),$tortuosity (B), and the critical diameter for cores taken from plots under different fertilizations and the woodland. The error bars represent standard errors.

Table 2 and Fig. 4A compare the porosity and pore-size distribution for the cores taken from different treatments respectively. Similar to their impact on aggregates, fertilization with farmyard manure and the woodland also simultaneously increased the porosity (pores > 40 μm) of the cores. Farmyard manure fertilization and the woodland increased proportions of the pores >0.5 mm at the expense of the pores smaller than 0.5 mm in the cores, compared to fertilization with inorganic fertilizers. However, contrary to the aggregates, the cores taken from the unfertilized plot had higher porosity than those fertilized with inorganic fertilizer, and they also had higher proportions of pores >0.8 mm than other treatments. Overall, fertilization with inorganic fertilizers appeared to have affected the pores in the cores more significantly than the intra-aggregate pores. Similar as in the aggregates, Fig. 4B shows that the porosity and tortuosity in the cores were also negatively correlated. Fig. 4C compares the critical pore diameter in both vertical and horizontal directions for all treatments. With no exception, the critical pore diameter in the horizontal direction is bigger than that in the vertical direction, especially for the farmyard manure treatment.

**Table 2:** Average and coefficient of variation (CV) of porosity and permeability of the cores under different treatments.

Treatment	Porosity		Vertical permeability (μm^2^)		Horizontal permeability (μm^2^)	
	Mean	CV	Mean	CV	Mean	CV
Woodland	0.375	0.0912	3386.43	0.2184	4002.6	0.2184
FYM	0.166	0.3476	110.87	0.6514	560.7	0.6514
N3	0.055	0.5275	8.31	1.9652	37.09	1.9652
No p	0.024	0.3865	0.42	1.4753	0.66	1.4753
Control	0.093	0.3733	43.87	1.0168	98.79	1.0168

### 3.2 Permeability of the aggregates

The three permeability components calculated from each aggregate differed from each other with their difference depending on aggregates and treatments. For all aggregates, the difference between the highest and lowest permeability components varied between 5% and 25%. As it was impossible to keep the orientation of the aggregates, in what follows we take the average of the three components of each sample as its permeability.

Table 1 compares the average permeability of the aggregates for the five treatments. Compared to the CK, fertilization with inorganic fertilizers did not result in a significant change in permeability, while fertilization with farmyard manure made the aggregates as permeable as returning the soil to natural woodland for more than one century. On average, increasing carbon input to the soil through farmyard manure application or perennial vegetation coverage as in the woodland improved aggregate permeability approximately fivefold from about 0.10 μm^2^ for treatments without exogenous carbon input to approximately 0.59 μm^2^ due to the increase in porosity and volumetric fraction of the intra-aggregate pores > 10.25 μm (Fig. 3A), as well as the reduced tortuosity (Fig. 3B). While the woodland treatment had more > 17.5 μm pores than other treatments, these pores were pore bodies linked by pore throats; they are important in retaining water but have limited contribution to soil permeability as proven in the pore-network models (Blunt et al., 2013). Applying N, P, K and Mg fertilizers did not appear to have altered the aggregate permeability noticeably compared to no-fertilization, because of their comparable pore-size distribution, porosity, tortuosity and critical pore diameter (Table 1, Fig. 3A – 3C).

The permeability varied with aggregates taken from the same treatment, and Fig. 3D compares the change in permeability with porosity for all aggregates. The porosity–permeability relationship for aggregates taken from plots unfertilized or fertilized with inorganic fertilizers follows approximately the same power law with *R*^2^ > 0.8 (Fig. 3D). In contrast, the permeability of the aggregates taken from farmyard manure and the woodland plots trends differently with their porosity (Fig. 3D), because of their difference in pore-size distribution and tortuosity (Fig. 3A, B).

Table 1 compares the coefficient of variation (CV) of the permeability - defined as the ratio of the standard deviation to the mean. The woodland aggregates were most homogenous with the least CV. Overall, the CV for the aggregates unfertilized or fertilized with inorganic fertilizer was higher than that for the aggregates taken from the farmyard manure and the woodland plots. Compared to no-fertilization and fertilization with N, K, P and Mg, fertilization without P since 2001 appeared to have made the aggregates more heterogeneous, with its CVs being 1.149 and larger than CV for all other treatments.

### 3.3 Permeability of the cores

Table 2 compares the average permeability components in both vertical and horizontal directions for all cores taken from the five treatments. We did not keep the horizontal orientation of the cores, and the horizontal permeability component in all tables and figures are the average of the two horizontal components calculated for each core. The permeability components in both vertical and horizontal directions of the cores taken from the farmyard manure and woodland plots were two to three orders of magnitude higher than that of other cores. The tortuosity of the aggregates unfertilized and fertilized with inorganic fertilizations was comparable (Fig. 3B), while the tortuosity of the cores under inorganic fertilizations was substantially higher than that in other treatments (Fig. 4B) due to the reduced porosity and the increased volumetric fraction of pores smaller than 0.4 mm (Fig. 4A). Fertilization without P since 2001 reduced the permeability of the cores, compared to those unfertilized or fertilized with N, K, P and Mg (Table 2), and the permeability of the cores taken from the woodland plot was much higher than that of the cores fertilized with farmyard manure.

All cores were hydraulically anisotropic with their permeability in the horizontal direction higher than that in the vertical direction, although the difference varied between treatments (Table 2). The cores in the woodland were most hydraulically homogeneous and isotropic, with the horizontal to vertical ratio being 1.3, while fertilization with farmyard manure made the soil most hydraulically anisotropic with the horizontal to vertical ratio increased by almost fivefold. Table 2 reveals that no-fertilization or fertilization with inorganic fertilizers made soil more hydraulically heterogenous at core scale, with their CVs being much higher than that in other treatments.

**Fig. 5:**
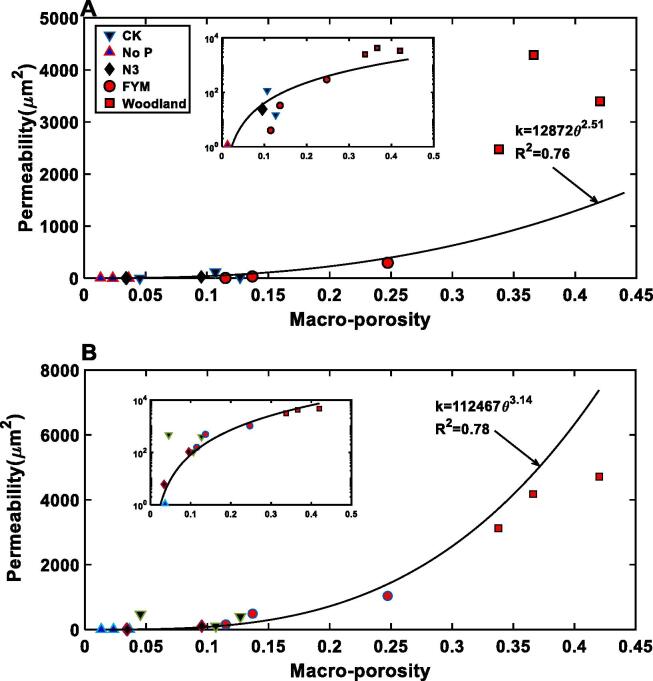
Macroporosity-permeability relationship for all cores taken from plots under different fertilizations and the woodland. Results for the vertical permeability component (A), and the horizontal component (B).

Fig. 5 shows the porosity - permeability relationship in both vertical and horizontal directions for the cores taken from all five treatments. Although they both followed approximately a power law, their *R*^2^ was smaller than that for the aggregates (Fig. 3D). As there were only three replicates for each treatment, we did not separately fit the core results for each treatment as we did for the aggregates. While the porosity and permeability of the cores taken from the cropped plots scattered around the power-law curves, the results of the woodland departed from the curves for both vertical and horizontal directions.

## 4 Discussion

The results obtained from the Rothamsted long-term experiment revealed that different fertilizations operating for 175 years had reshaped the soil structure differently, and that the structural change varied with scales. In all but the fertilization without P since 2001, the permeability of the cores was one to three orders of magnitude higher than that of the aggregates. The ability of the soils to infiltrate water at saturation following rainfalls was thus dominated by the pores>40 μm as found at this experimental site in 1882 (Beven and Germann, 1982), which was also corroborated by results obtained from X-ray images of other soils (Katuwal et al., 2015, Paradelo et al., 2016, Zhang et al., 2019).

### 4.1 The cores

The permeability of the cores varied significantly between the treatments due to their difference in porosity, the number of hydraulically connected large macropores (Table 2, Fig. 4A) and the tortuosity (Fig. 4B). As expected, the permeability of the cores in the farmyard manure and the woodland plots was much higher than that in other plots, because the exogenous carbon input enhanced soil aggregation and increased the proportion of pores > 0.5 mm (Fig. 4A). This is consistent with work reported by others (Dal Ferro et al., 2013, Yang et al., 2018). For the woodland, non-tillage is another mechanism underlying the increased core porosity and permeability as non-tillage kept the macropores generated by roots and soil fauna intact (Borges et al., 2019, Galdos et al., 2019), especially the pores > 1.2 mm (Fig. 4A).

It was unexpected that non-fertilization increased the porosity and volumetric fraction of pores > 0.75 mm at the expense of the pores  smaller than 0.7 mm, thereby increasing soil permeability, compared to fertilization with inorganic fertilizers. It is unclear that this unexpected result was due to the limited replicated samples, which were insufficient to be representative to the heterogeneous field soil, or that this was indeed the structural change in the field. Increasing soil samples could resolve this uncertainty, but there was a trade-off between costs and accuracy as three replicates are the minimum requirement for the results to be statistically meaningful and have been widely adopted in experimental design (Dal Ferro et al., 2013). Notwithstanding that, as the critical diameter of the unfertilized cores was only 0.4 mm (in the vertical direction) and 0.8 mm (in the horizontal direction), most large pores were pore bodies linked by small pore throats and played only a limited role in conducting water. Therefore, its permeability was still much smaller than that of the farmyard manure and woodland cores.

The permeability of the cores fertilized without P since 2001 was the lowest in both vertical and horizontal directions (Table 2 and Fig. 5). This was due to the combined impact of the reduced porosity (Table 2) and the increased tortuosity, rather than the change in proportion of different pores as the pore-size distributions of the cores fertilized with and without P were comparable (Fig. 4A). A decrease in hydraulically connected large pores could reduce soil permeability substantially as the permeability increases with pore diameter parabolically. Despite the cores fertilized with and without P having comparable tortuosity and pore size distribution (Fig. 4A-B), the critical pore diameter of the former (0.057 mm) was much smaller than that of the latter (0.34 mm) (Fig. 4C), which, along with their difference in porosity, was the key reason behind the reduced permeability of soil without P application. As the average permeability of a series of connected pores in a hydraulic conduit is the geometric mean of the permeability of these pores, the contribution of large pore bodies linked by smaller pore throats to soil permeability is minor although they increase the ability of soil to hold water (Li et al., 2018a).

Cropping appeared to have made the cores hydraulically anisotropic with their horizontal permeability component much higher than their vertical component, although the level of the anisotropy varied with treatments (Table 2). There is no consensus in the literature about how cropping systems impact soil anisotropy, with some showing isotropy while others showing anisotropy. However, it is generally accepted that peat soils are more homogenous than arable soils (Gharedaghloo et al., 2018), which is consistent with our result although the underlying mechanisms remain obscure. The woodland on the experimental site has grown naturally since 1882, and it could be because of the accrual of rooting activities and other biotic and abiotic processes that had made the cores relatively isotropic and homogeneous (Table 2).

**Fig. 6:**
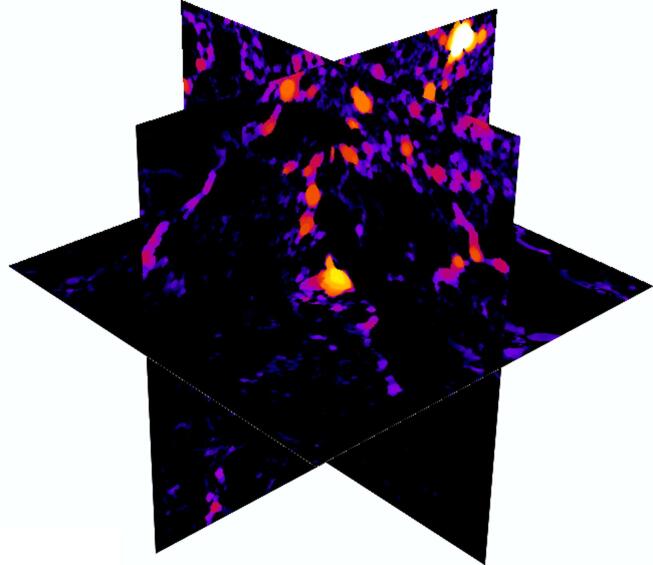
An illustrative example showing the spatial locations of pores of different sizes in a core receiving farmyard manure where the large pores are more abundant in the top than in the bottom (Black represents matrix, and the pore size increases from dark to bright).

The cropping field is ploughed annually after harvest with a mouldboard plow, and the crop husbandry was the same in all treatments. Therefore, the hydraulic anisotropy was likely to be caused by fertilization-induced abiotic and biotic processes, among which earthworm burrowing could be an important one. Field surveys since 1922 have consistently shown that the number of earthworms in the plots continuously receiving farmyard manure was significantly higher than those in other treatments. For example, a 2014 survey found that the total earthworm biomass in plots given farmyard manure was 109 g/m^2^, compared to 6 g/m^2^ in the plots never receiving farmyard manure (Sizmur et al., 2017). In all treatments, the endogeic earthworm was found to be the dominant species (Sizmur et al., 2017) which is known to make horizontal burrows (Le Couteulx et al., 2015), proliferating in topsoil (Schlüter et al., 2018). This was reflected in pore geometry, as Fig. 4C shows that the more hydraulically anisotropic the cores are, the more their critical pore diameters in the vertical and horizontal directions differ from each other. As an illustration, Fig. 6 shows the locations of all pores in a farmyard manure core where large pores are more abundant in the top (topsoil) than in the bottom (subsoil).

The pores in the cores are not only hydrologically important for providing quick pathways for fluid to flow through, they are also biologically critical for modulating nutrient cycling as well as root growth (Atkinson et al., 2020). An increase in porosity and permeability of the cores could lead to a rise in yield due to the increase in nitrification and root penetration as our previous work based on the Rothamsted long-term experiments showed that more porous and permeable soils were positively related to abundance of nitrification-related genes (Neal et al., 2020b). The average yield of the plots given farmyard manure, inorganic fertilizer and the CK was 11 t/ha, 5.5 t/ha and 1.2 t/ha, respectively. The yield is hence positively related to the porosity and permeability of the cores (Table 2), except the CK due to the lack of nutrients input.

### 4.2 The aggregates

Soil aggregates are formed largely through binding of soil particles by fungus and decomposed organic matter, and its life expectancy is much longer than the macropores (Rabot et al., 2018). The comparatively isotropic aggregates suggested that the genesis of the aggregate structure was impacted less directly by root than by microbially-mediated processes powered by rhizodeposits and other organic matter. Adding carbon boosts microbial activities thereby enhancing soil aggregation and increasing porosity of fine textured soil (Helliwell et al., 2014), but the increase varied with pore size. For the farmyard manure aggregates, it was the pores in 10 μm − 20 μm that increased most, while for the woodland aggregates the pores > 12.5 μm were relatively more abundant (Fig. 3A). The experimental site was rich in fungus and the total bacterial community in the woodland was twofold that in the arable soil (Hargreaves et al., 2003). These could be an important mechanism underlying the increased porosity (Fig. 3A) and permeability (Table 1) of the aggregates in the farmyard manure and the woodland plots (Crawford et al., 2012).

The tortuosity of the pores in the aggregates was lower than that of the cores subjected to the same treatment, indicating that the pores in the former were less tortuous for water to flow than in the latter. In general, pore connectivity increases with porosity and our results revealed that the aggregates are more porously connected than the cores. This is contrary to the results of Dal Ferro et al (2013) who found that their long-term fertilizations (since 1962) made pores in cores more connected than the intra-aggregate pores in a field cropped with maize. While it cannot be ruled out that different crops could have different impacts on multiscale soil structure, the difference in duration between the experiments might also play a role. Also, the low image resolution (6.25 μm) used by Dal Ferro (2013) could be a reason as it was not high enough to capture the dominant pores to make the images representative to the real aggregates. This manifests the importance of imaging resolution in interpreting how soil structure evolves after a management change.

There is a subtle difference in how permeability of the aggregates varied with their porosity between the treatments (Fig. 3C). The porosity–permeability relationship for the aggregates unfertilized or fertilized with inorganic fertilizers followed approximately the same trend, alluding that the pore geometries in these aggregates were statistically similar and that the impact of the crop on aggregate structure might have overridden the impact of the fertilizations. In contrast, the permeability of the aggregates in the farmyard manure and the woodland plots increased with their porosity in a different trend, indicating that the pore geometries in their aggregates were statistically different from that in others and that the farmyard manure impacted the intra-aggregate structure more than the crop did. This is corroborated by the pore-size distribution in Fig. 3A where the proportion of pores > 10 μm in the farmyard manure and its associated critical pore diameter (Fig. 3D) are much higher than those in other fertilization treatments.

### 4.3 Impact of soil carbon

**Fig. 7:**
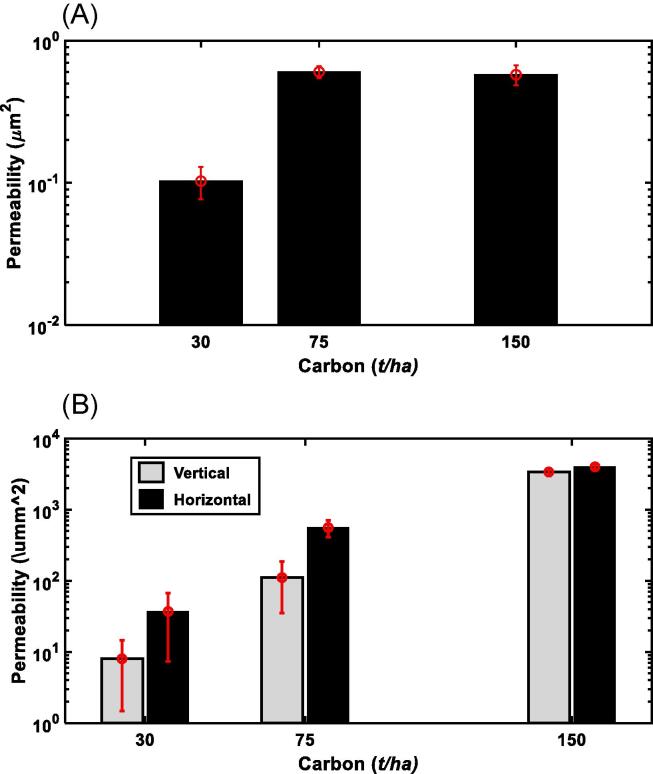
The increase in permeability with soil carbon at aggregate scale (A), and core scale (B).

Soil structural change is affected by many abiotic and biotic factors including root growth and macro- and *meso*-fauna. Increasing carbon input usually leads to an increase in porosity and ability of the soil to hold and to transport water, and it is thus interesting to see how permeability of the aggregates and cores responded to soil carbon change. The total carbon in the top 23 cm of soil in the plots given farmyard manure has increased from 30 t/ha in1843 to 75 t/ha, while in the woodland it has increased to 160 t/ha. In contrast, the carbon in the plots fertilized only with mineral fertilizers remained almost unchanged while the carbon in the top 23 cm of soil not receiving any inputs reduced to 25 t/ha. Fig. 7A shows that with soil carbon increasing, the permeability of the aggregates increased asymptotically plateauing when the carbon exceeded 75 t/ha, while the permeability of the cores, especially its vertical component, increased exponentially (Fig. 7B). Such increases in permeability with carbon at both scales were due to the increase in total porosity and the volumetric fraction of hydraulically connected large pores: pores>12 μm for aggregates (Fig. 3A) and pores>0.8 mm for the cores (Fig. 4A); these combined to reduce the tortuosity and the resistance for water to flow because of the increase in critical pore diameter at both scales (Fig. 3, Fig. 4). Fig. 7 indicates that as soil carbon increased, the impact of carbon on pores > 40 μm in the cores was more significant than on the intra-aggregate pores, consistent with recent findings that soil enzymatic activities, which modulate soil carbon distribution, are not randomly distributed but closely associated with pores in a specific range (Kravchenko et al., 2019). Given the increasing interest in sequestering carbon into soil, understanding how soil at different scales hydraulically responds to carbon input is important but appears to have been overlooked (Poulton et al., 2018).

## 5 Conclusions

Multiscale X-ray imaging combined with lattice Boltzmann simulation reveals that different fertilizations operating continuously for more than one century have reshaped the soil structure differently, and the soil structural change and its relationship with soil carbon vary with scales. Increasing carbon input to soil via farmyard manure application has increased porosity and permeability at both aggregate and core scales, and the hydraulic properties of their aggregates are very close to that of the aggregates in the woodland that has been returned to nature since 1882. In all treatments, the aggregates are approximately hydraulically isotropic while the cores are anisotropic with their permeability component in the horizontal direction being higher than that in the vertical direction, although the level of the anisotropy varies between treatments. The porosity–permeability relationships are similar for aggregates in soils unfertilized or fertilized with inorganic fertilizers, indicating that the crop might overweight the fertilization in their impact on aggregate structure. In contrast, the permeability of the aggregates in the farmyard manure and the woodland plots trends differently with their porosity.

An increase in carbon input to soil via farmyard application or perennial vegetation has made the soil more porous and permeable, but the change in hydraulic properties with soil carbon varies with scale. As soil carbon increases, the permeability of the cores, especially its vertical component, increases exponentially, while the permeability of the aggregates increases asymptotically - peaking when the carbon reaches a critical value. We also found a positive correlation between soil permeability and crop yield.

## CRediT authorship contribution statement

**Xiaoxian Zhang:** Formal analysis, Investigation, Software, Visualization, Writing - original draft. **Andrew L. Neal:** Data curation, Methodology. **John W. Aurelie Crawford Bacq-Labreuil:** Project administration, Methodology, Data curation, Visualization. **Elsy Akkari:** Methodology, Data curation. **William Richard:** Data curation.

## Declaration of Competing Interest

The authors declare that they have no known competing financial interests or personal relationships that could have appeared to influence the work reported in this paper.
