# Supplementary material for: The effects of long-term fertilizations on soil hydraulic properties vary with scales
Source: J Hydrol (Amst). 2021 Feb;593:125890. doi: 10.1016/j.jhydrol.2020.125890 (PMC7871028; doi:10.1016/j.jhydrol.2020.125890)
Supplement: Supplementary Data 1 [file mmc1.docx]

**Supplementary information**

**S1. Fertilization history of the sampled plots**

The soils were sampled in October 2015 from four treatments at the Broadbalk long-term field experiment (51°48′35″ N, 00°22′30″ W, Johnston and Poulton, 2018).


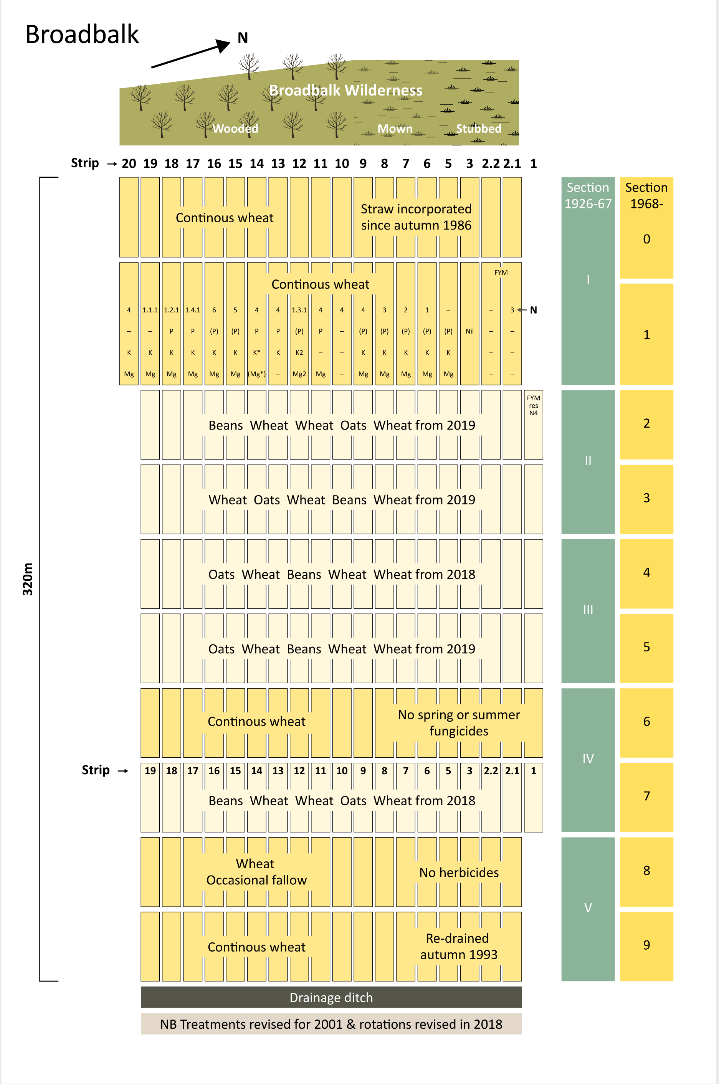


Figure S1. The long-term wheat experiment at Rothamsted

The experimental soil is a clay loam to silty clay loam over clay with flints (FAO Classification: Chromic Luvisol) and is slightly calcareous. It is inclined slightly toward the east and is under free drainage by subsurface drain buried underneath each plot. The drained water from each drain is collected from an open trench at the east end of the experimental site as shown in Figure S1. The four treatment plots are on Section 1 on the west side of the experimental site as shown in Figure S1, which has been sown continuously with winter wheat (*Triticum aestivum* L., most recently Crusoe seed coated with Redigo® Deter® combination insecticide/fungicide treatment, Bayer CropScience) since 1843, except for occasional fallow years to control weeds. Triplicate samples were taken from each of the four plots: Plot 3 -1 (Strip-Section) that has not received fertilization since 1843 **(referred to as CK)**. Plot 2.2-1 that has been fertilized with composted farmyard manure (from cattle) applied at a rate of 35 t ha^-1^ per year since 1843 **(referred to as FYM)**. Plot 8-1 (**referred as N3**) that has received complete inorganic fertilizer (fertilizer^+NP^) containing 144 kg ha^-1^ nitrogen (N), 35 kg ha^-1^ phosphorus (P) as triple superphosphate (calcium dihydrogen phosphate), 90 kg ha^-1^ potassium (K) as potassium sulfate, and 12 kg ha^-1^ magnesium (Mg) as kieserite per year since 1852. Plot 20-1 that received inorganic 96 kg ha^-1^ N as ammonium sulphate, 35 kg ha^-1^ P, 90 kg ha^-1^ K and 12 kg ha^-1^ Mg per year (fertilizer^-N^) since 1906. Nitrogenous fertilizer was applied to fertilizer^+NP^ and fertilizer^-P^ soils as ammonium sulfate until 1967, calcium ammonium nitrate (Nitro-chalk) 1968–1985 and as Nitram® ammonium nitrate since 1986. Since 2001, Plot 20-1 has not received P as it was considered in excess (referred to as **No P**). The plough layer (0–23 cm) is limed when necessary - due to increasing soil acidity largely resulting from long-term use of ammonium sulfate as a source of N on some plots - to maintain a minimum soil pH of 7.0–7.5. Liming began in Autumn 1954, and a total chalk application of 18.4 and 10.4 t ha^-1^ was applied to fertilizer^+NP^ and fertilizer^-P^ soils respectively, up until 1974. No chalk was applied to the other soils. From 1975 to 1989 a regular scheme was introduced, and a total of 14.7 t ha^-1^ chalk was applied to each soil. No further chalk was required until 2007. Since then, fertilizer^+NP^ soil received a total of 6 t ha^-1^ chalk up to 2015; the other three plots did not require chalk over this period. All soils are tilled conventionally. Since treatments are not replicated on the field experiment, three *pseudo*-replicates were collected from each treatment plot. These *pseudo*-replicates were collected from each end and the centre of the plot, approximately 9 m apart. The local mean annual temperature and rainfall are 10.1 ^o^C and 701 mm, respectively. The soil is predominantly clay loam classified as Chromic Luvisol (FAO classification).

**S2. The lattice Boltzmann model**

Water flow in all imaged samples was simulated using the lattice Boltzmann (LB) method by tracking the movement and collision of a number of fictitious fluid particles under constraints that their movement and collision conserve mass and momentum. The propagation of each particle in the pore space in each sample is described by the following multiple-relaxation time lattice Boltzmann equation (d'Humieres et al., 2002):

$f_{i}\left(\text{x}+\delta t\text{e}_{i},t+\delta t\right)=f_{i}\left(\text{x},t\right)+M^{-1}SM\left[f_{i}^{eq}\left(\text{x},t\right)-f_{i}\left(\text{x},t\right)\right],$ (S1)

where$f_{i}\left(\text{x},t\right)$is the particle distribution function at location ***x*** and time *t* moving at lattice velocity ***e****_i_*, δ*x* is the size of the voxels in the image, δ*t* is time step, $f_{i}^{eq}\left(\text{x},t\right)$ is equilibrium distribution function, *M* is a transform matrix and *S* is the collision matrix. The product M*f* in Eq. (A1) transforms the particle distribution functions to a moment space in which the collision operation of$m=SM\left[f_{i}^{eq}\left(\text{x},t\right)-f_{i}\left(\text{x},t\right)\right]$ is performed. The post-collision results in the moment space are then transformed back to particle distribution functions by$M^{-1}m$. We use the D3Q19 lattice model in this paper in which the particle distribution functions move in 19 directions at 19 velocities: $\left(0,\text{ }0,\text{ }0\right)$, $\left(\pm\delta x/\delta t,\text{ }\pm\delta x/\delta t,\text{ }0\right)$, $\left(0,\text{ }\pm\delta x/\delta t,\text{ }\pm\delta x/\delta t\right)$, $\left(\pm\delta x/\delta t,\text{ }0,\text{ }\pm\delta x/\delta t\right)$and $\left(\pm\delta x/\delta t,\text{ }\pm\delta x/\delta t,\text{ }\pm\delta x/\delta t\right)$(Qian et al., 1992). The collision matrix is diagonal the terms in which are

$\begin{matrix} S=\left(s_{0},s_{1},s_{2},s_{3},s_{4},s_{5},s_{6},s_{7},s_{8},s_{9},s_{10},s_{11},s_{12},s_{13},s_{14},s_{15},s_{16},s_{17},s_{18}\right)^{T}, \\ s_{0}=s_{3}=s_{5}=s_{7}=0, \\ s_{1}=s_{2}=s_{9-15}=1/\tau, \\ s_{4}=s_{6}=s_{8}=s_{16-18}=8(2-\tau^{-1})/(8-\tau^{-1}), \end{matrix}$ (S2)

The fluid simulated by the above model has a kinematic viscosity$\mu=\delta x^{2}(\tau-0.5)/3\delta t$ and pressure$p=\rho\delta x^{2}/3\delta t^{2}$. The equilibrium distribution functions in Eq. (A1) are defined as follows:

$\begin{matrix} f_{i}^{eq}=w_{i}\left[\rho+\rho_{0}\left(\frac{3\text{e}_{i}\cdot\text{u}}{c^{2}}+\frac{9\left(\text{e}_{i}\cdot\text{u}\right)^{2}}{2c^{4}}-\frac{3\text{u}\cdot\text{u}}{2c^{2}}\right)\right], \\ w_{0}=1/3, \\ w_{i}=1/18,\text{ }\left\|\text{e}_{\text{i}}\right\|=\delta x/\delta t \\ w_{i}\text{=}1/36\text{ }\left\|\text{e}_{\text{i}}\right\|=\sqrt{2}\delta x/\delta t\text{ } \end{matrix}$ (S3)

where $c=\delta x/\delta t$ and $\rho_{0}$is a reference fluid density to ensure that the fluid is incompressible when the flow is in steady state (Zou et al., 1995). The bulk fluid density ρ and velocity ***u*** are updated after each time step from

$\begin{matrix} \rho=\sum_{i=0}^{18} f_{i}, \\ \rho_{0}\text{u}=\sum_{i=1}^{18} f_{i}\text{e}_{i}. \end{matrix}$ (S4)

Implementation of the above model needs two steps to advance a time step. The first one is to calculate the collision in the moment space and then transform the results back to particle distribution functions, i.e., to calculate$f_{i}^{*}=f_{i}\left(\text{x},t\right)+M^{-1}SM\left[f_{i}^{eq}\left(\text{x},t\right)-f_{i}\left(\text{x},t\right)\right]$; and the second step is to move the post-collision particular distribution function$f_{i}^{*}$ to position at $\text{x}+\delta t\text{e}_{i}$during the time period of δ*t*. During the streaming step, whenever$f_{i}^{*}$hits a solid voxel, it is bounced back to where it was before the streaming to represent a non-slip boundary where bulk fluid velocity is zero.

Fluid flow in an image was driven by a pressure gradient applied in a direction, generated by imposing two different pressures on two opposite sides of the image in that direction. We then simulated the flow to steady state and once the flow was deemed to have reached steady state, fluid velocity and pressure in all voxels were sampled and used to calculate the permeability in this reaction from Eqs. (S2) and (S3). We then applied the pressure gradient into other two orthogonal directions to calculate the permeability components in other directions.

**References**

d'Humieres, D., Ginzburg, I., Krafczyk, M., Lallemand, P., Luo, L.S., 2002. Multiple-relaxation-time lattice Boltzmann models in three dimensions. Philos. Trans. R. Soc. Lond. Ser. A-Math. Phys. Eng. Sci., 360(1792): 437-451. DOI:10.1098/rsta.2001.0955

Qian, Y.H., Dhumieres, D., Lallemand, P., 1992. Lattice BGK models for Navier-Stokes equation. Europhysics Letters, 17(6BIS): 479-484. DOI:10.1209/0295-5075/17/6/001

Zou, Q.S., Hou, S.L., Chen, S.Y., Doolen, G.D., 1995. An improved incompressible Lattice Boltzmann model for time-independent flows. . Journal of Statistical Physics, 81(1-2): 35-48. DOI:10.1007/bf02179966
